# Accuracy in detecting major depressive episodes in older adults using the Swedish versions of the GDS-15 and PHQ-9

**DOI:** 10.48101/ujms.v126.7848

**Published:** 2021-10-20

**Authors:** Johnny Pellas, Mattias Damberg

**Affiliations:** a Department of Public Health and Caring Sciences, Uppsala University, Uppsala, Sweden; b Centre for Clinical Research, Uppsala University, Västmanland County Hospital, Västerås, Sweden

**Keywords:** Depression, geriatric, validation, screening, rating scales

## Abstract

**Objectives:**

The purpose of this study was to evaluate the diagnostic accuracy at different cut-off values for the Swedish versions of the 15-item Geriatric Depression Scale (GDS-15) and Patient Health Questionnaire (PHQ-9) compared with a structured clinical psychiatric interview in older adults.

**Methods:**

Community-dwelling participants (*N* = 113) aged 65 years or older completed the Swedish versions of the GDS-15 and PHQ-9 and were then interviewed using the Mini International Neuropsychiatric Interview (MINI) to establish the presence or absence of current major depressive episodes (MDEs). Areas under the curve (AUC) were calculated for each scale, as well as the sensitivity, specificity, and Youden’s index for different cut-off values.

**Results:**

Seventeen participants met the criteria for MDEs. The AUC was 0.97 for the GDS-15 and 0.95 for the PHQ-9. A cut-off of ≥6 on the GDS-15 yielded a sensitivity of 94%, a specificity of 88%, and a Youden’s index of 0.82. A cut-off of ≥5 on the PHQ-9 yielded a sensitivity of 100%, a specificity of 81%, and a Youden’s index of 0.81. The proposed cut-off of ≥10 on the PHQ-9 produced excellent specificity of 95% but a lower sensitivity of 71%.

**Conclusions:**

This study indicates that the Swedish versions of the GDS-15 and PHQ-9 have comparable accuracy as screening instruments for older adults with MDEs. However, the proposed cut-off of 10 on the PHQ-9 might be too high when applied to older individuals in Sweden, and further investigations in larger samples in different healthcare settings are warranted.

## Introduction

In Sweden, the prevalence of depression in adults aged 60 years or above is about 6% in community settings (1) and 15% in primary care (2). Depression in older adults increases the risk of mortality and morbidity (3), leads to functional impairments (4), and reduces quality-of-life (5) and is therefore important to identify and treat. Using depression questionnaires may increase the depression detection rate and thereby increase the number of people with depression receiving treatment (6). The Swedish Agency for Health Technology Assessment and Assessment of Social Services recommends the use of the 15-item Geriatric Depression Scale (GDS-15) for depression screening in older adults (7, 8) and the Patient Health Questionnaire (PHQ-9) for adults in general (9, 10).

The GDS was originally a 30-item questionnaire for depression screening in older adults but was modified into a shorter questionnaire consisting of 15 items (11). A meta-analysis revealed a sensitivity of 89% and a specificity of 77% for the GDS-15 with a cut-off of >5 (12). The GDS-15 has been translated into Swedish, and a 20-item version, the GDS-20, has also been developed, which includes additional items of insomnia, anxiety, panic, pain, and somatization (13). The Swedish GDS-15 has acceptable reliability and validity at different levels of cognitive functioning (14). A study by Sacuiu and colleagues (15) reported a sensitivity of 71% and a specificity of 93% when using a cut-off of 9, but the diagnosis was made using another rating scale, and the sample consisted of older individuals who had made a suicide attempt 1 year before and thus constituted a psychiatric sample that may not apply to individuals living in community settings.

The PHQ-9 is a nine-item screening questionnaire used to identify depression and to measure its severity (16). A meta-analysis revealed a sensitivity of 88% and a specificity of 78% at a cut-off of 10 or above (9). The Swedish PHQ-9 has acceptable reliability and validity (17).

There are, to our knowledge, no published studies that have compared the Swedish versions of the PHQ-9 and GDS-15 in older individuals and no reports of the diagnostic accuracy that have used a structured clinical interview as a reference test for the Swedish versions of PHQ-9 or GDS-15 in older adults. Therefore, the aim of the present study was to evaluate the diagnostic accuracy at different cut-off values for the Swedish GDS-15 and PHQ-9 compared with a structured clinical interview in older adults.

## Materials and methods

### Design

The present study was a retrospective, cross-sectional diagnostic accuracy study. The sample was a convenience sample pooled from two separate trials: 1) *N* = 77 participants were included from the Psychiatric Syndromes in Late Life – Assessment and Treatment Study, PLLAT, a trial aimed at validating Swedish versions of psychiatric measures, using data collected in 2019–2020, and 2) *N* = 36 participants were included from the CoviDep-study, a trial of telephone-based psychological treatment for depressive symptoms in older individuals in isolation during COVID-19, using data collected in 2020 (ClinicalTrials ID NCT04508868).

Both studies received ethical approval from the Swedish Ethical Review Authority (registration numbers 2019-00944 and 2020-02079). All participants were recruited from the County of Västmanland in Sweden and were residing in the community. All participants provided written informed consent.

### Participants

The participants were approached through organizations for senior citizens in the County of Västmanland as well as via advertisements in local newspapers. The inclusion criteria were 1) 65 years old or above, 2) fluent in spoken and written Swedish, and 3) willing to participate in the trial. For the CoviDep-study, the participants also experienced low mood and/or diminished interest in activities. The exclusion criteria for both the trials were a current substance use disorder, current diagnosis of dementia/major neurocognitive disorder, and current diagnosis of a neurological condition or severe visual impairment (not able to read the questionnaires). In the PLLAT-study, there was also a lower limit of 25 points on the cognitive screening test Mini-Mental State Examination, which could not be applied in the CoviDep-trial because all contact was by telephone.

### Materials

#### Mini International Neuropsychiatric Interview

The Mini International Neuropsychiatric Interview 7.0 (MINI) (18), a structured clinical interview, was used as a reference test to assess the presence or absence of major depressive episodes (MDEs) and/or other common psychiatric disorders according to the *Diagnostic and Statistical Manual of Mental Disorders* 5th Edition (*DSM-5*). The MINI has high reliability and validity (18) and a sensitivity of 95% and a specificity of 84% compared with the Structured Clinical Interview for DSM-IV-Axis-I Disorders (SCID-I) (9).

#### Geriatric Depression Rating Scale 15

The GDS-15 (11) is a questionnaire used to identify depression in older individuals with scores ranging from 0 to 15.

#### Patient Health Questionnaire-9

The PHQ-9-item is a questionnaire used to identify depression and its severity, with scores ranging from 0 to 27, with higher scores indicating higher depression severity (16).

### Procedure

The procedure differed between the two samples because of the COVID-19 pandemic. The participants from the PLLAT-trial came to the study research clinic and filled out the rating scales. They were then interviewed by a clinical psychologist (demographic data and MINI) on the same day. Participants from the CoviDep-trial performed rating scales at home and were interviewed by a clinical psychologist by telephone (demographic data and MINI). Only the participants who were interviewed within 2 weeks of performing the rating scales were included from the CoviDep-trial. A research nurse scored the rating scales to ensure that the psychologists were blinded to the results. All interviews in the PLLAT-trial were performed by the same psychologist (the corresponding author), whereas additionally four psychologists performed the interviews in the CoviDep-trial. All psychologists were trained in administering the MINI. The diagnosis of depression was made using the MINI algorithm for current MDEs, according to the *DSM-5*.

### Analyses plan

Diagnostic accuracy was calculated with sensitivity and specificity for different cut-off values, as well as the area under the curve (AUC). We chose 70% as the minimum level of sensitivity and specificity. Optimal cut-off values were determined using Youden’s index (sensitivity + specificity – 1).

## Results

**Figure 1:**
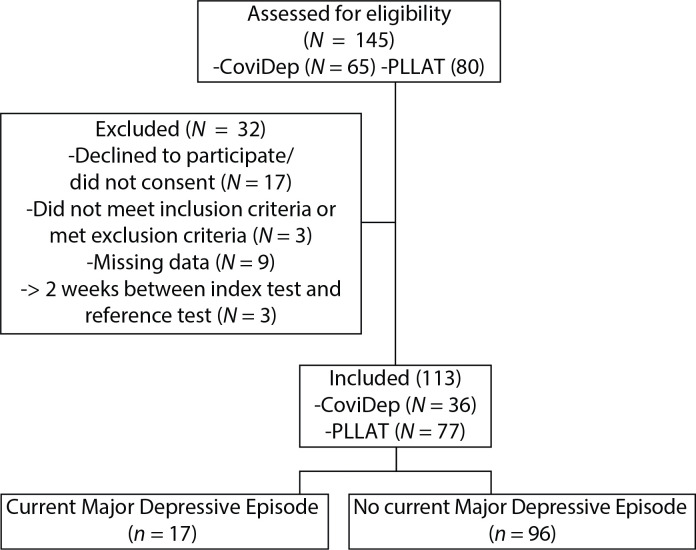
Participant flow.

A total of 113 participants were included in the study. Participant flow is described in Figure 1. Based on the diagnostic procedure, 17 participants were classified as having a current MDE. Baseline demographic and clinical characteristics are documented in Table 1. None of the participants were receiving specialized psychiatric care.

**Table 1:** Demographic and clinical characteristics.

	Total sample (*N* = 113)
Age, mean (SD) years	75.65 (6.1)
Women, *n* (%)	83 (73.5)
Major Depressive Episode, *n* (%)	17 (15)
GDS-15, mean (SD)	3.24 (3.8)
PHQ-9, mean (SD)	4.39 (5.5)

SD: standard deviation; GDS-15: Geriatric Depression Rating Scale 15-item short form; PHQ-9: Patient Health Questionnaire 9.

The AUC was 0.97 for the GDS-15 and 0.95 for the PHQ-9. Sensitivity and specificity values for different cut-off points are shown in Table 2, illustrating the cut-off values with at least 70% sensitivity and 70% specificity, as well as the proposed cut-off values. According to Youden’s index, the optimal cut-off was 6 points and above for the GDS-15 and 5 points and above for the PHQ-9 prioritising sensitivity. Cross tabulation for the proposed cut-off value of ≥6 for the GDS-15 appears in Table 3, for the proposed cut-off value of ≥10 for the PHQ-9 in Table 4, and for the optimal cut-off value of ≥5 for the PHQ-9 in Table 5.

**Table 2:** Sensitivity, specificity and Youden’s index of GDS-15 and PHQ-9 at different cut-off points.

Instrument and cut-off point	Sensitivity (%) (CI)	Specificity (%) (CI)	Youden’s index
GDS-15			
≥4	100 (80–100)	76 (66–84)	0.76
≥5	100 (80–100)	81 (72–88)	0.81
*≥6*	***94 (71–100)***	***88 (79–93)***	***0.82***
≥7	88 (64–99)	91 (83–96)	0.79
≥8	82 (57–96)	93 (86–97)	0.75
≥9	71 (44–90)	97 (91–99)	0.68
PHQ-9			
≥4	100 (80–100)	72 (62–81)	0.72
**≥5**	**100 (80–100)**	**81 (72–88)**	**0.81**
≥6	88 (64–99)	83 (74–90)	0.71
≥7	88 (64–99)	86 (78–93)	0.74
≥8	88 (64–99)	93 (86–97)	0.81
≥9	82 (57–96)	93 (86–97)	0.75
*≥10*	*71 (44–90)*	*95 (88–98)*	*0.66*

GDS-15: Geriatric Depression Rating Scale 15-item short form; PHQ-9: Patient Health Questionnaire 9; CI: 95% confidence interval.

*Note*. Bold cut-off values indicate the optimal balance of sensitivity and specificity based on Youden’s index prioritising sensitivity, whereas italicized cut-off values represent the proposed cut-off values.

**Table 3:** Cross tabulation of Major Depressive Episode and the Geriatric Depression Scale 15 at ≥6 points.

GDS-15	Major Depressive Episode		Total
	Yes	No	
≥6	16	12	28
<6	1	84	85
Total	17	96	113

GDS-15: Geriatric Depression Rating Scale 15-item short form.

**Table 4:** Cross tabulation of Major Depressive Episode and the Patient Health Questionnaire 9 at ≥10 points.

PHQ-9	Major Depressive Episode		Total
	Yes	No	
≥10	12	5	17
<10	5	91	96
Total	17	96	113

PHQ-9: Patient Health Questionnaire 9.

**Table 5:** Cross tabulation of Major Depressive Episode and the Patient Health Questionnaire 9 at ≥5 points.

PHQ-9	Major Depressive Episode		Total
	Yes	No	
≥6	17	18	35
<6	0	78	78
Total	17	96	113

PHQ-9: Patient Health Questionnaire 9.

## Discussion

This is, to our knowledge, the first study to compare the diagnostic accuracy of the Swedish GDS-15 and PHQ-9 in older adults and the first study to use a structured clinical interview as a reference standard for these tests in Swedish. The results indicate that the GDS-15 and PHQ-9 have comparable diagnostic accuracy in classifying older adults with MDEs. However, the proposed cut-off of 10 on the PHQ-9 might be too high for the application to older adults in Sweden, a conclusion in line with studies of older adults in other countries that have reported an optimal cut-off of 6 (19, 20), and despite other studies that have found the proposed cut-off value to be optimal (21). Our findings highlight the importance of further studies of the appropriate cut-off on the PHQ-9 because it is widely used and recommended for use in primary health care in Sweden (10). The difference between the cut-off values in different countries may reflect cultural differences but may also reflect the use of different settings, populations, and age groups. In this study, none of the participants received psychiatric care and were all recruited from the community.

There are several limitations to this study. Firstly, the total sample was pooled from two trials with differences in the procedure; 77 participants performed the MINI face-to-face directly after filling in the rating scales, whereas 36 participants filled in the rating scales at home and performed the MINI over the telephone within 2 weeks. However, the MINI has been found to produce equivalent results when administered via the telephone compared with in-person interview (22), and a maximum of 2 weeks between the index test and reference test has been allowed in a recent meta-analysis of the GDS (12). Secondly, in the PLLAT-trial, all interviews were administered by the same psychologist, whereas additionally four psychologists administered the interviews in the CoviDep-trial. Although all psychologists were experienced in administering the MINI and the interview is highly structured, we did not investigate the inter-rater reliability. Thirdly, the samples differed in that the CoviDep-participants were recruited for a depression treatment trial and thereby subjectively depressed, whereas the PLLAT-subjects were not recruited based on subjective feelings of depression. This might contribute to a spectrum effect and was accounted for by including participants with subclinical depressive symptoms in the control group and not excluding participants with other psychiatric conditions. None of the participants were receiving specialized psychiatric care, suggesting that no cases of more severe depression were included. Finally, although using a structured clinical interview as a reference test is considered a strength in diagnostic accuracy studies, it might be considered potentially problematic when used with older individuals because the symptoms in depression might differ from those in younger and middle-aged adults, with older adults more often fulfilling the criteria for minor depression than major depression (7). There is, however, no consensus about the differences in symptoms of depression between younger and middle-aged adults and older adults (23), nor is there any consensus on specific diagnostic criteria for depression in older individuals. Nonetheless, future studies could use, for example, a diagnostic procedure based on the Longitudinal, Expert, All Data-procedure as a reference standard, as suggested by others (7).

In summary, our study indicates that the Swedish versions of the GDS-15 and PHQ-9 are viable options for case finding of MDEs in older adults. However, while the cut-off of ≥6 on the GDS-15 seems optimal, the cut-off on the PHQ-9 may need to be lowered to ≥5 instead of ≥10. Further studies are needed to evaluate the accuracy of the GDS-15 and PHQ-9 for older adults in different care settings.

## Data access

Data are not available because of legal issues.
